# A laid-back trip through the Hennigian Forests

**DOI:** 10.7717/peerj.3578

**Published:** 2017-07-21

**Authors:** Evgeny V. Mavrodiev, Christopher Dell, Laura Schroder

**Affiliations:** 1 University of Florida, Florida Museum of Natural History, Gainesville, FL, USA; 2 Department of Anatomy and Neurobiology of University of Tennessee, University of Tennessee Health Science Center Washington, Memphis, TN, USA; 3 Washington, Wyoming, Alaska, Montana and Idaho Medical Education Program, University of Idaho, Moscow, ID, USA

**Keywords:** Binary and multistate characters, Cladograms, Matrix-free cladistics, Maximal relationship, Character polarity, Synapomorphy, Supertrees, Binary and multistate characters, Average consensus method, Standard maximum parsimony analysis

## Abstract

**Background:**

This paper is a comment on the idea of matrix-free Cladistics. Demonstration of this idea’s efficiency is a major goal of the study. Within the proposed framework, the ordinary (phenetic) matrix is necessary only as “source” of Hennigian trees, not as a primary subject of the analysis. Switching from the matrix-based thinking to the matrix-free Cladistic approach clearly reveals that optimizations of the character-state changes are related not to the real processes, but to the form of the data representation.

**Methods:**

We focused our study on the binary data. We wrote the simple ruby-based script FORESTER version 1.0 that helps represent a binary matrix as an array of the rooted trees (as a “Hennigian forest”). The binary representations of the genomic (DNA) data have been made by script *1001*. The Average Consensus method as well as the standard Maximum Parsimony (MP) approach has been used to analyze the data.

**Principle findings:**

The binary matrix may be easily re-written as a set of rooted trees (*maximal* relationships). The latter might be analyzed by the Average Consensus method. Paradoxically, this method, if applied to the Hennigian forests, *in principle* can help to identify clades *despite* the absence of the direct evidence from the primary data. Our approach may handle the clock- or non clock-like matrices, as well as the hypothetical, molecular or morphological data.

**Discussion:**

Our proposal clearly differs from the numerous phenetic alignment-free techniques of the construction of the phylogenetic trees. Dealing with the relations, not with the actual “data” also distinguishes our approach from all optimization-based methods, if the optimization is defined as a way to reconstruct the sequences of the character-state changes on a tree, either the standard alignment-based techniques or the “direct” alignment-free procedure. We are not viewing our recent framework as an alternative to the three-taxon statement analysis (3TA), but there are two major differences between our recent proposal and the 3TA, as originally designed and implemented: (1) the 3TA deals with the three-taxon statements or minimal relationships. According to the logic of 3TA, the set of the minimal trees must be established as a binary matrix and used as an input for the parsimony program. In this paper, we operate directly with maximal relationships written just as trees, not as binary matrices, while also using the Average Consensus method instead of the MP analysis. The solely ‘reversal’-based groups can always be found by our method without the separate scoring of the putative reversals before analyses.

## Introduction

Here it would seem more appropriate to re-write the characters …. in a tree-form representing the relationships exactly

Williams & Ebach (2006: 414)

... synapomorphies might evolve more than once. Suppose, indeed, that all synapomorphies really have multiple origins. Would our understanding of relationships then be any different than it now is?

Nelson (2011: 140)

Matrix-free Cladistics (Platnick, 1993; Williams & Ebach, 2006; Williams & Ebach, 2008; Zaraguëta-Bagils et al., 2012) is a way to represent the binary, morphological and molecular data in tree form, therefore dispensing with the traditional (ordinary) matrix. Demonstration of this idea’s efficiency is a major goal of the study.

Recently, the matrix-free Cladistic approach has been developed by Zaraguëta-Bagils et al. (2012). Our framework, however, is different from their proposal, which we discussed below. In general, we are viewing the method developed by Zaraguëta-Bagils et al. (2012) as their original conceptualization of the three-taxon statement analysis (3TA) (Nelson & Platnick, 1991) which is essentially based on the principle of the maximum compatibility (see Wilkinson, 1994a for the initial discussion).

One may note that our vision of the topic replaces the standard “processes-based” subject with a purely geometric view of the problem. Another may see that our viewpoint is closely related to the old discussions around “Transformed cladistics” (Platnick, 1979; Platnick, 1985) or “pattern-cladistics”, or “Cladistics” (with the capital “C”, see for example Hull, 1988). Numerous works have been published on topic in the past (e.g., Betty, 1982; Brady, 1982; De Queiroz & Donoghue, 1990; Nelson, 1989; Nelson & Platnick, 1981; Patterson, 1980; Patterson, 1982; Platnick, 1979; Platnick, 1982; Platnick, 1985 see also Brady, 1985; Brower, 2000; Eldredge & Cracraft , 1980; Ebach & Williams, 2013; Nelson, 1972; for the detailed reviews of the topic see Hull, 1988; Farris, 2014, Scott-Ram, 1990 and especially Williams & Ebach, 2008). We would like the reader to make his own conclusions regarding the actual summary of these mostly aged but still inspiring debates, which in our minds, were terminated artificially. Of course, it would be impossible to reproduce anything but the shadow of that story in this Introduction.

However, today it is still critical to repeat that dealing with the ‘trees’ (or with the statements of relationships) can be viewed separately from any considerations of the process (e.g., Kitching et al., 1998: p. 1; Patterson, 1980: p. 239). Also, the statements of the relationships may be easily treated just *as invariants* of the processes (either real or hypothetical). For example, when considering the statement A(BC) it is easy to conclude that it does not really matter what the actual cause or process that linked taxa B and C altogether was.

We agree that the consistent implementation of the basic idea of Hennig—grouping solely on synapomorphies returns us to purely comparative thinking (e.g., Nelson, 2004; Williams & Ebach, 2008), even in the case of molecular data. From that, the treatment of the standard optimization procedures, performed either under the maximum parsimony or other criteria, as something that is related to the reality with its processes, appears to be very naive. In our mind, switching from the matrix-based thinking to the matrix-free Cladistic approach clearly reveals that the optimizations of the character-state changes are related not to the reality *per se*, but to the form of the data representation.

The aspects of the Cladistics we approved are clearly summarized in Patterson (1980):

“*Hennig’s 1966 book, as the title Phylogenetic Systematics suggests, was based in evolutionary theory…But as the theory of cladistics has developed, it has been realized that more and more of the evolutionary framework is inessential, and may be dropped. The chief symptom of this change is the significance attached to nodes in cladograms. In Hennig’s book, as in all early work in cladistics, the nodes are taken to represent ancestral species. This assumption has been found to be unnecessary, even misleading, and may be dropped. Platnick (1979) refers to the new theory as “transformed cladistics” and the transformation is away from dependence on evolutionary theory. Indeed, Gareth Nelson, who is chiefly responsible for the transformation, put it like this in a letter to me this summer: “In a way, I think we are merely rediscovering pre-evolutionary systematics, or if not rediscovering it, fleshing it out*” ”

(Patterson, 1980: p. 239; italics ours).

As Nelson himself defined later, Cladistics is “*ordering homologies into a parsimonious hierarchy, which specifies both taxa and their characters (apomorphies)*” (Nelson, 1989: 279), and therefore it is independent from the principle of common descent (Nelson, 1989).

Among others, De Queiroz & Donoghue (1990) have criticized this vision of the topic. In our mind, their most important contra-arguments are:

(1) Nelson’s vision of cladistics is strongly related to the “model of nested hierarchy”, even if they are supposedly independent of the principle of common descent (De Queiroz & Donoghue, 1990: p. 62);
(2) the conventional phylogenetic systematics has greater explanatory power than that which underlies what Nelson calls “cladistics” (De Queiroz & Donoghue, 1990: p. 61, see also Farris et al., 1995 and Farris, 2013).

The later criticism of the transformed cladistics has been developed by James Farris and his school (see Farris et al., 1995 and Farris, 1997; Farris, 2011; Farris, 2012; Farris, 2014 for the discussions and the summaries of the arguments), essentially in relation to the possible issues of the three-taxon statement analysis (Nelson & Platnick, 1991), that we, contrary to Brower (2000), are viewing as the first analytical implementation of the transformed cladistics approach (e.g., Kitching et al., 1998). Below we show that the major empirical claims against 3TA, established by Farris and co-authors (see Siebert & Williams, 1998, Mavrodiev, 2015b and Mavrodiev, 2016 for the reviews), are not relevant to our recent framework.

In our mind, the whole focus of the “explanatory power” (e.g., De Queiroz & Donoghue, 1990; Farris et al., 1995) is essentially misleading due to its strong relation to the particular philosophy of science that different authors are following or implying. For example, this focus is not quite relevant within the framework of critical philosophy (Mavrodiev, 2016, see also Van de Vijver et al., 2005 and Kolen & Van de Vijver, 2007 among others), which has likely never been mentioned in the discussed context until recently.

### A. Summary of used and implied concepts with selected references and clarifying citations:

a. An ordinary (phenetic) matrix and matrix-free Cladistics (Platnick, 1993; Williams & Ebach, 2006; Williams & Ebach, 2008);
  *“The notion that systematic data constitute a normal characters x taxon matrix is not an intrinsically cladistic notion; indeed, that type of matrix seems to have originated with pheneticists. Consider an alternative view, that the three-taxon matrix instead constitute systematic data…*” (Platnick, 1993: p. 271; italics ours).
  “The data matrix can be generally viewed in three different ways for systematics and biogeography, which we refer to as phylo-phenetics, phylogenetic systematics (transformational) and Cladistic. *Each views the matrix as a combination of manipulating points (taxa, areas and characters) and cells (character states), expressed in a table. All current methods, except Cladistics, treat the transformations or switches between cells and points as the basis for discovering and expressing relationships …for most methods the data matrix is simply a phenetic device for optimising homologues* rather than determining homologies and discovering relationships”
  (Williams & Ebach, 2006: p. 409; italics ours).
b. The symmetry between binary character and cladogram, as conceptualized by Williams (1994 and 1996);
  “*The binary character (as represented in a column in a matrix) and the cladogram (as represented by branching diagram) are one and the same thing*, representations of relationships”
  (Williams, 1994: pp. 451–452, italics ours).
c. Maximal relationship (Nelson & Platnick, 1981), summarized in Williams & Ebach (2006) and Williams & Ebach (2008), see also Wilkinson (1994b: p. 344–345).
  “…the component (11) is only a “part” of the data (the 00 is a part too), hence to express a specific relationship, both aspects of the data require consideration. Thus, the relationship is AB(CD). *This relationship can be thought of as “maximal” (after (Nelson & Platnick, 1981), in that it includes all the taxic points* (A–D)”.
  (Williams & Ebach, 2006: p. 412, italics ours).
d. Homology as a relationship (summarized in Williams & Ebach, 2008 and Nelson, 2011);
  “*For Owen homology is a relation between homologues, not merely the homologues themselves*; and the same for analogy and analogues. For him, the relation between homologues means that the homologues are represented in an archetype, the concept that does the relating (Williams, 2004: 196). For vertebrates, his archetype is an ideal (hypothetical) vertebra, or a series of vertebrae and their associated nerves and muscles (cf. Panchen, 1994). Hence the relation is that of “similar to” or “same as” an archetype”.
  (Nelson, 2011: p. 137, italics ours).
e. Outgroup comparison (Wiley, 1976, see also Platnick & Gertsch, 1976, Watrous & Wheeler , 1981 and Nixon & Carpenter, 1993 for the review);
  “Hennig’s (1966) method differs fundamentally from a purely phenetic method in that all the shared characters are not used to refute a given relationship; only synapomorphous characters are used. Such testing can be accomplished only in an open system, that is, by considering taxa outside the three (or more) taxon system. Such considerations may be termed outgroup comparisons”
  (Wiley, 1976: p. 11).
  “To determine which of two or more homologous states is primitive and which derived, we have used two sources of evidence, immediate outgroup comparison and ontogeny. . . The use of outgroup comparison requires knowledge of the closest relative of the entire group under consideration, which in this case we suggest is the arachnid order Amblypygi. The hypothesis that spiders and amblypygids are sister groups is supported by at least two apparently autapomorphic characters: they are the only arachnids with subchelate chelicerae and with both a pumping pharynx and a pumping stomach . . . We thus hypothesize that any character state found in some but not all spiders and also in amblypygids is plesiomorphic, and its homologs apomorphic; this hypothesis can be falsified in any particular case by incongruence with more numerous synapomorphy patterns. . .
  (Platnick & Gertsch, 1976: p. 2).
f. *A priori* determination of character’s polarity (reviewed in Kitching et al., 1998 and Waegele, 2005; see also Williams & Ebach, 2008 and Wiley & Lieberman , 2011);
  “…*when coding polarized characters for cladistics analyses* all plesiomorphic character-states should be marked with a “0” in the data matrix, all apomorphies (when only two character-states are present) with “1”…”
  (Waegele, 2005: p. 183; italics ours).
g. Grouping solely on synapomorphy (Hennig, 1966; see also Wiley, 1975, Wiley, 1976, Platnick, 1985, Nelson, 2004, Williams & Ebach, 2006 and Williams & Ebach, 2008 among others);
  “That a common stem form is shared by a group of species (a condition for a monophyletic group) *can be proven only by means of synapomorphous characters, not by symplesiomorphous characters* ”
  (Hennig, 1966: p. 90; italics ours).
h. Cladistics is more than Wagner’s algorithm or its optimization-based derivatives (Nelson, 2004; Williams & Ebach, 2006; Williams & Ebach, 2008);
  “For Hennig, “…*we can never directly observe the phylogenetic transformation of a character*”. (Summarized by Nelson, 2004: p. 134; italics ours).
i. Average Consensus method (Lapointe & Cucumel, 1997; Lapointe & Levasseur, 2004) and its extensions (Creevey, 2004, see also Lapointe & Levasseur, 2004);
  “The average consensus method combines the information from multiple trees by calculating the path length from every taxa to every other taxa on each of the source trees. This method utilizes branch lengths (if present) or assumes a branch length of unity (a branch length of one) if not present …The specifics of the method are as follows:
  • The distance from each taxon to every other taxon on a tree is calculated. This is the equivalent of the sum of the branch lengths in the path between the two taxa.
  • The average distance of each taxa to every other taxa across all the source trees is calculated…
  • A least squares method is used to estimate a supetree phylogeny (with branch lengths) that best describes the distance matrix …You also have the option of doing a neighbor joining supertree from the calculated distance matrix” (Creevey, 2004: p. 18).
j. Matrix representation with Parsimony (Baum, 1992; Ragan, 1992);
  “Rooted phylogenetic trees can be represented as matrices in which the rows correspond to termini, and columns correspond to internal nodes (elements of the *n*-tree). Parsimony analysis of such a matrix will fully recover the topology of the original tree”
  (Ragan, 1992: p. 53).
k. Three-taxon (item) statement analysis (3TA) (Nelson & Platnick, 1991); see also Kitching et al., 1998 and Williams & Ebach, 2008 for the reviews.
  “Three-item statement analysis. A method of cladistic analysis that focuses on the smallest unit of relationship, the three-item statement, rather than on characters. The observed features of taxa are coded in terms of the relationships they imply, that is three-item statements, and the optimal cladogram is that which maximizes the number of accommodated three-item statements”
  (Kitching et al., 1998: p. 218).

### B. Initial propositions

A binary character is a tree with one informative node (Platnick et al., 1996; Williams, 1994; Williams & Ebach, 2006; Williams & Ebach, 2008). For example, if state 1 is apomorphic, then the character ABCDE/00011 *is a rooted tree* ABC(DE), where (DE) is a clade (monophyletic group) based on the apomorphic character-state. If all of the clades of the tree are based on apomorphic character-states, we call this tree a “Hennigian” tree. If we accept that the trees such as ABC(DE) or A(BC(DE)) are Hennigian, than the trees A((BC)(DE)) or ((ABC)(DE)) are not. The non-Hennigian trees contain groups based on plesiomorphic characters-states—for example, tree ((ABC)(DE)) is non-Hennigian because group (ABC) based on the plesiomorphic characters-state zero. Hennigian trees may also be easily seen as simple hierarchies of two character-states.

Let A be defined as the outgroup. In this case the *relationship* ABC(DE) may be re-written as A(BC(DE)) (or as a (A(BC(DE)))), *even if, strictly speaking, we do not have any formal evidence for the groups* (BCDE) (or (ABCDE))v (see Rieppel, 1991: p. 95 and Williams & Ebach, 2009 for the related discussions). For example, the *minimal relationship* A(BC)/011 may be re-written as (A(BC)), even if there is no evidence for the group (ABC).

Consider the tree A(BC(DE)) for one more time. If the value of taxon B is missing, two solutions appear to be possible.

First, character ABCDE can be re-written as two trees, assuming that the missing value may be either zero or one: ABC(DE) = A(BC(DE)) or A(C(BDE)). Another possibility implies the exclusion of the taxon B from the tree. This reduces the character ABCDE to the tree AC(DE).

Characters like ABCDE/00001, 000?1, ??001, 00000 etc. collapse to polytomies such as (ABCDE), (ABCE) etc.

The basic idea of Matrix Representation with Parsimony (Baum, 1992; Ragan, 1992) states that the tree (cladogram) can be represented as a binary matrix. Here we propose something opposite—we accept that the binary character may be re-written as a tree (Nelson & Ladiges, 1992, 492–493; Williams, 1994; Siebert & Williams, 1998: 342; Williams & Ebach, 2006: 414; see also Platnick et al., 1996). Therefore, the binary matrix can be viewed as a set (forest, array etc.) of branching diagrams (cladograms) that may be called a “Hennigian forest” if one of the character-states (for example, the character-state zero) *a priori* is defined as plesiomorphic.

It is easy to assume that the Hennigian forest might be analyzed at least by some consensus methods, typically treated as a ways of estimating the supertrees (Gordon, 1986, see also Wilkinson, Cotton & Thorley, 2004 and Bininda-Emonds, 2014 for the reviews) for example, by the Average Consensus method (Lapointe & Cucumel, 1997; Lapointe & Levasseur, 2004, see also Bininda-Emonds, 2014). Using this method as an example, below we are attempting to display the workings of this assumption.

As summarized by Lapointe & Levasseur (2004: 87), the Average Consensus procedure is a method that takes as input a profile of weighted trees (i.e., trees with the branch lengths Lapointe & Cucumel, 1997; Creevey, 2004; Lapointe & Levasseur, 2004) and returns a consensus tree that is, in some sense, “closest” to the entire profile. Originally this method was designed to combine the clock-like or “ultrametric” rooted trees (or the trees with the total branch length from the root up to any tip equal (Felsenstein, 2004: p. 161)), but later have been extended to allow for the combination of all types of the weighted trees, the clock-like or not (reviewed in Lapointe & Levasseur, 2004: 88 and Creevey, 2004: p. 18).

This method allows us to operate with the Hennigian trees directly, completely excluding the binary data matrix from the analysis. The ordinary (phenetic) matrix remains necessary only as a “source” of the forest of the Hennigian trees, not as a primary subject of the analysis. Within this framework, the criteria of the best trees, such as the minimal numberof character-state changes, as well as the standard optimization procedures, if optimization is defined as a way to reconstruct the sequences of the character-state changes on a tree, all appeared to be unrequired.

The matrix-free Cladistic approach, as designed and implemented by Zaraguëta-Bagils et al. (2012) and others (reviewed in Zaraguëta-Bagils et al., 2012), also treats the characters as hierarchies or as rooted trees, but clearly differs from our proposal in several aspects. According to the logic and implementation of Zaraguëta-Bagils et al. (2012), the rooted character-state trees must first be reduced to a set of three-item statements. The software LisBeth (Zaraguëta-Bagils et al., 2012) performs the modified three-taxon statement analysis, which is based on the maximum congruence of the statements and finally calculates the intersection that the tree is built from, and only from, the three-taxon statements that are common to all the optimal trees and characters (Zaraguëta-Bagils et al., 2012).

Our approach also differs *in principle* from the numerous purely phenetic alignment-free techniques of the construction of the phylogenetic trees (reviewed and summarized in Warnow, 2014 and Bogusz & Whelan, 2016) because these methods do not even refer to the concepts of the “relation” and “homology”, but operate by computing similarities (the pairwise distances) between the raw (or non-aligned) sequences (e.g., Warnow, 2014).

Finally, one may ask why the data cannot be imputed directly as trees (Zaraguëta-Bagils et al., 2012) or, in other words, by just completely skipping the data matrix? In principle, it is possible. However so far this solution seemed for us to be impractical in many cases.

**Figure 1:**
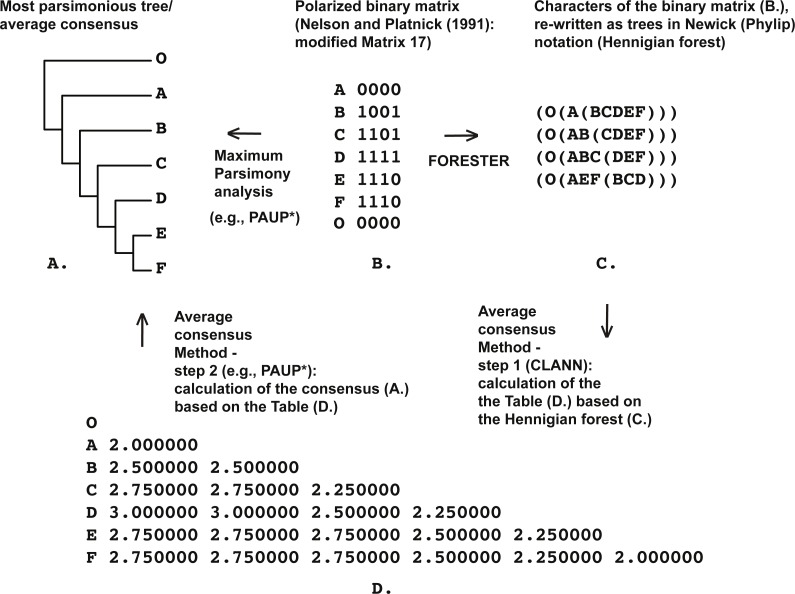
(A) Most parsimonious tree of the length is equal to five (CI = 0.80, RI = 0.86) based on the Matrix 17 (B) from Nelson & Platnick (1991: p. 362) with character four excluded, tree was a posteriori rooted relatively taxon O∖the average consensus tree of the score 0.22222 calculated based on the table (D); (B) Matrix 17 from Nelson & Platnick (1991: p. 362) with the character four excluded; (C) four remaining characters of the Matrix 17 (B) established as Hennigian trees; (D) the average consensus table based on the forest (C).

## Materials and Methods

Despite the plethora of publications within the field of contemporary Bioinformatics, *there is no software available to rewrite the binary matrix as a Hennigian forest*. Given this, we wrote the simple ruby-based script FORESTER version 1.0 (named “FORESTER” below) that helps represent the binary data as Hennigian trees for future manipulations (Figs. 1–9).

FORESTER (deposited on https://github.com/dellch/forester) processes each input file by storing the text of each line of the file as an element of an array. It finds the beginning and ending location (index) of the characters in the matrix, and loops from the starting index to the ending index, writing a line of the new file(s) at the end of each loop.

For the binary Matrix, the usage of the script is: **ruby trees.rb –inputfilename**

The pre-defined out-group taxon should be placed last in the matrix before running of the FORESTER.

Three output tree-files are available as a result of the run: the first file contains polytomies such as (ABCDE)(the output tree-file named as a *“With poly…”* ), while the second and the third outputs appear without such polytomies, but trees may be rooted relative to *a priori*-defined outgroup taxon (e.g., (A(BC(DE)))) (the output array appears as named as *“No poly…”* tree-file) or to the basal polytomy (e.g., (ABC(DE))) (the output tree-file named as an *“Additional…”* tree-file).

All tree files and the input binaries should be written in the “relaxed” non-interleaved Newick (PHYLIP) format (reviewed in Felsenstein, 1989, see also Maddison & Maddison, 2011 and Swofford, 2002).

The minimal trees (the three-taxon statements (3TS)) are not the subjects of our recent considerations, but FORESTER contains options for speedy rewriting of the 3TS matrices as the arrays of the minimal trees. The usage should be: **ruby seedlings.rb –inputfilename** with only one output Newick file saved as a result of the run.

**Figure 2:**
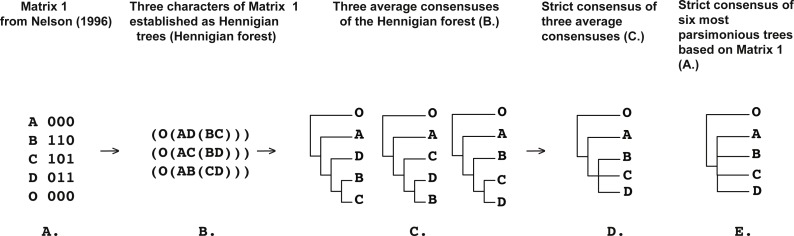
(A) Matrix 1 modified from Nelson (1996: p. 1) (Nelson, 1996; Williams & Ebach, 2005); (B) three conflicting characters of Matrix 1 from Nelson (1996: p. 1) (A) (see also Williams & Ebach, 2005) established as a Hennigian trees; (C) three average consensuses of the forest (B) of the score zero and their strict consensus (D); (E) strict consensus of six most parsimonious trees of the length equal to five (CI = 0.6000, RI = 0.3333) based on the Matrix 1 from Nelson (1996: p. 1). All six trees were *a posteriori* rooted relatively taxon O.

**Figure 3:**
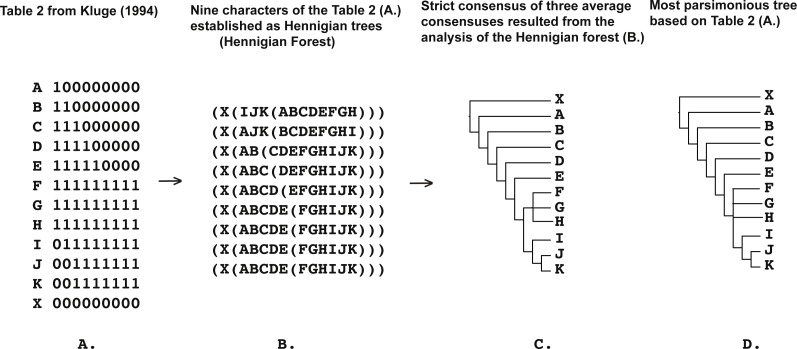
(A) Table 2 modified from Kluge (1994: p. 408); (B) nine characters of the Table 2 from Kluge (1994: p. 408) (A) established as a Hennigian trees; (C) strict consensus of three average consensuses of the score 0.27118 resulted the analysis of the forest (B); (D) most parsimonious tree of the length = 11 (CI = 0.8182, RI = 0.9429) based on the Table 2 from Kluge (1994: p. 408). Tree was *a posteriori* rooted relatively taxon X.

**Figure 4:**
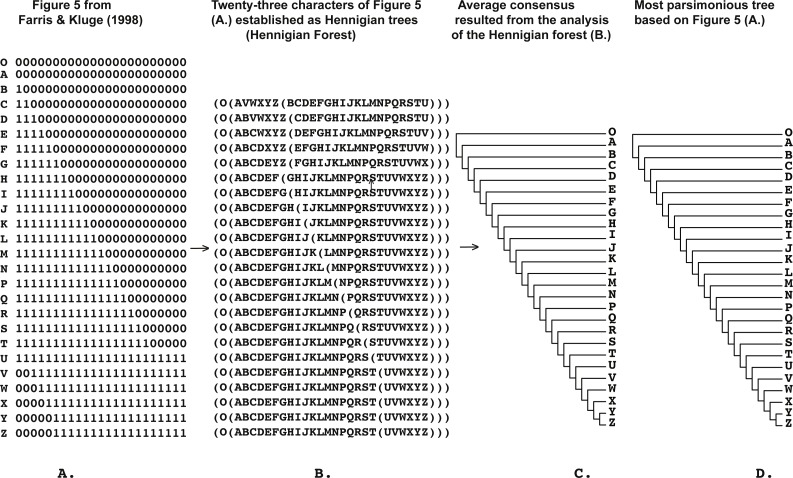
(A) Figure 5 modified from Farris & Kluge (1998: p. 353); (B) 23 characters of Figure 5 modified from Farris & Kluge (1998: p. 353) (A) established as a Hennigian trees; (C) the average consensus tree of the score 1.00184 resulted the analysis of the forest (B); (D) most parsimonious tree of the length = 28 (CI = 0.8214, RI = 0.9711) based on the Table 2 from Farris & Kluge (1998: p. 353). Tree was *a posteriori* rooted relatively taxon O.

**Figure 5:**
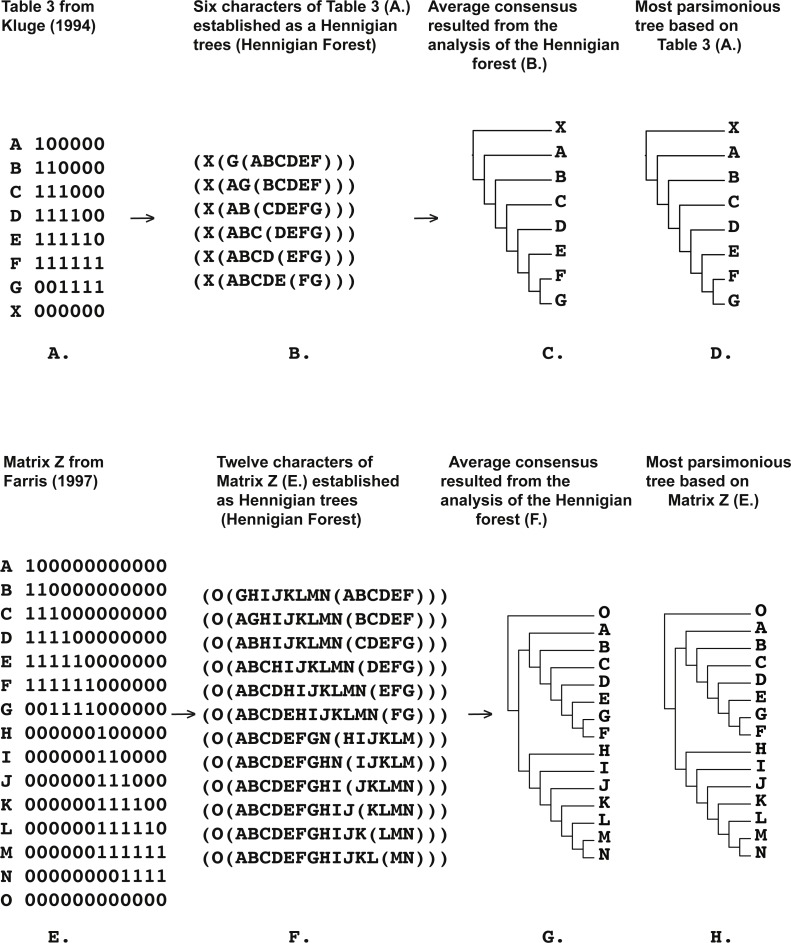
(A) Table 3 modified from Kluge (1994: p. 409); (B) six characters of Table 3 from Kluge (1994: p. 409) (A) established as a Hennigian trees; (C) the average consensus tree of the score 0.29213 resulted the analysis of the forest (B); (D) most parsimonious tree of the length is equal to eight (CI = 0.7500, RI = 0.8182) based on the Table 3 from Kluge (1994: p. 408). Tree was *a posteriori* rooted relatively taxon X; (E) Matrix Z from Farris (1997: p. 136); (F) 12 characters of Matrix Z from Farris (1997: p. 136) (A) established as a Hennigian trees; (G) the average consensus tree of the score 1.9603e–08 resulted the analysis of the forest (F); (H) most parsimonious tree of the length = 16 (CI = 0.7500, RI = 0.8947) based of Matrix Z from Farris (1997: p. 136). Tree was *a posteriori* rooted relatively taxon O.

Seven binary matrices (Figs. 1–4, 5, 9) are taken from the literature (Nelson, 1996; Kluge, 1994; Farris, 1997; Farris & Kluge, 1998; Kluge & Farris, 1999).

**Figure 6:**
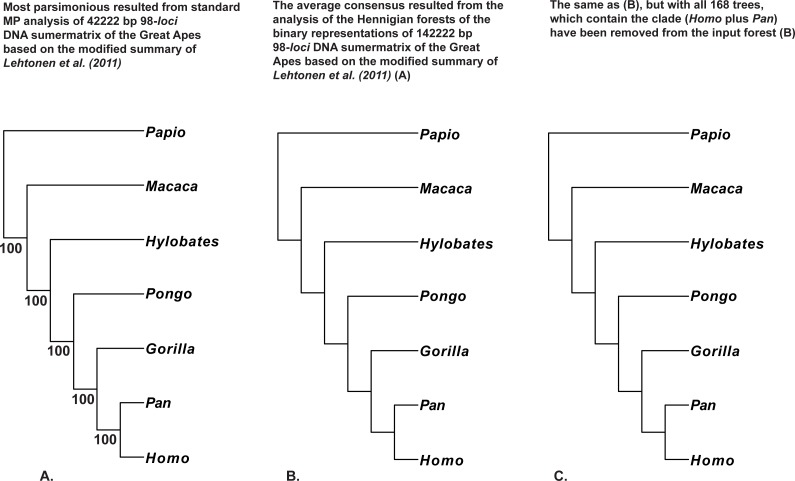
(A) Tree resulted the standard MP analysis of the 98-*loci* supermatrix of the Great Apes (Hominidae, Primates, Mammalia) constructed using the modified summary of Lehtonen et al. (2011) (see “Materials and Methods” for the details) and *a posteriori* rooted relatively *Papio* (length = 12,092, CI = 0.7877, RI = 0.5597); 34,022 characters are constant, and the number of the parsimony-informative characters is equal to 4,311. MP BS values are provided below branches. The only characters with non-ambiguous value of the outgroup (*Papio*) have been saved within concatenated alignment; (B) the average consensus tree of the score 0.00084 resulted the analysis of the Hennigian forest of the 5,507 trees derived from the binary representation of the 98-*loci* supermatrix of the Great Apes (Hominidae, Primates, Mammalia) (see “Materials and Methods” for the details); *Papio* is assumed to be the best all-plesiomorphic group. “Additional…” FORESTER’s output tree file (see “Materials and Methods” for the details) selected for future analyses; (C) the average consensus tree of the score 0.00090 resulted the analysis of the Hennigian forest of the 5,339 trees derived from the modified binary representation of the 98-*loci* supermatrix of the Great Apes (Hominidae, Primates, Mammalia) (see **6.I.** and “Materials and Methods” for the details), but with all 168 trees, which contain the clade (Homo plus Pan) have been removed from the input forest. “Additional…” FORESTER’s output tree file (see “Materials and Methods”) selected for future analyses.

Preparing the DNA supermatrix of the multiple loci of the Great Apes (Hominidae, Primates, Mammalia) (Fig. 6) we first used MUSCLE (Edgar, 2004) to align separate 98 gene regions listed in the modified summary of the Lehtonen et al. (2011). The resulting separate DNA alignments were concatenated in the SeaView 4.0 (Gouy, Guindon & Gascuel, 2010) after removing purely aligned regions using the Gblocks 0.91 (Talavera & Castresana, 2007) as implemented in a Seaview (Gouy, Guindon & Gascuel, 2010). The Gblocks less stringent selection strategy (Talavera & Castresana , 2007) was decided to be the best choice. Eventually, the only characters with non-ambiguous value of the outgroup (*Papio*) have been saved within concatenated 42,222 bp alignment.

**Figure 7:**
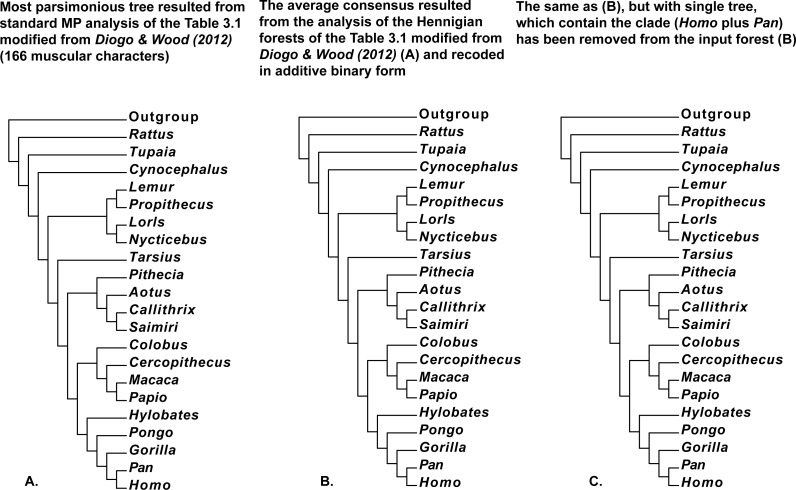
(A) Tree resulted standard MP analysis of modified Table 3.1 with 166 muscular characters of Primates (Mammalia) and *Rattus* (Mammalia, Rodentia, Muridae) from Diogo & Wood (2012: p. 121), *a posteriori* rooted relatively added artificial all-zero taxon (length = 307, CI = 0.5700, RI = 0.7295). 50 characters are parsimony-uninformative, and the number of the parsimony-informative characters is equal to 116. All multistate characters (60, 68, 124, 129, 136, 138, 149, and 162) have been treated as ordered; (B) the average consensus tree of the score 23.83826 resulted the analysis of the Hennigian forest of the 123 trees derived from the Table 3.1 modified from Diogo & Wood (2012: p. 121) with all multistate characters recoded in additive binary form; (C) the average consensus tree of the score 23.84628 resulted the analysis of the Hennigian forest of the 122 trees derived from complete Table 3.1 modified from Diogo & Wood (2012: p. 121) with all multistate characters recoded in additive binary form, but with the single tree with the clade (*Homo* plus *Pan*) has been removed from the input forest.

Matrix of 166 muscular characters of Primates (Mammalia) and *Rattus* (Mammalia, Rodentia, Muridae) has been modified from the image of Diogo & Wood (2012: p. 121, Table 3.1). We treated all multistate characters from Table 3.1 (Diogo & Wood (2012: p. 121) as ordered (reviewed in Kitching et al., 1998) (Fig. 7) and recoded all of them in additive binary form (reviewed in Kitching et al., 1998) using TAXODIUM ver. 1.2 (Mavrodiev & Madorsky, 2012).

**Figure 8:**
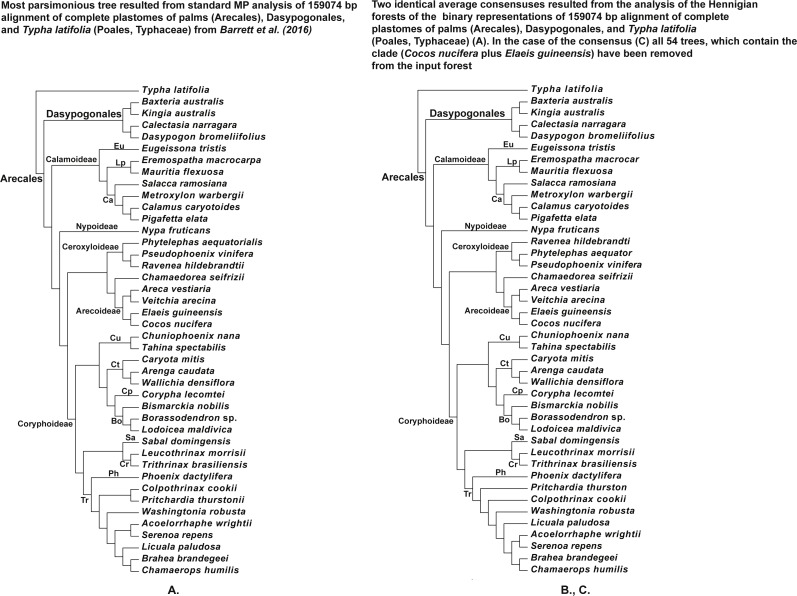
(A) Tree resulted standard MP analysis of 159,074 bp alignment of complete plastomes of palms (Arecales), Dasypogonales, and *Typha latifolia* (Poales, Typhaceae) from Barrett et al. (2016) (see their Fig. 4 and Fig. S5), a posteriori rooted relatively the cattail (*Typha*) (length = 66,788, CI = 0.7363, RI = 0.6109), 119,251 characters are constant, and the number of the parsimonyinformative characters is equal to 19,124; (B) the average consensus tree of the score 0.00755 resulted the analysis of the Hennigian forest of the 25,630 trees derived from the modified binary representation of the original 159,074 bp alignment of complete plastomes of palms (Arecales), Dasypogonales, and *Typha latifolia* (Poales, Typhaceae) from Barrett et al. (2016) (see “Materials and Methods” for the details). The cattail is assumed to be the best all-plesiomorphic group. “Additional…” FORESTER’s output tree file (see “Materials and Methods” for the details) selected for future analysis; (C) the average consensus tree of the score 0.00760 resulted the analysis of the Hennigian forest of the 25,576 trees, derived from the binary representation of the original 15,9074 bp alignment of the complete plastomes of palms (Arecales), Dasypogonales, and *Typha latifolia* (Poales, Typhaceae) from Barrett et al. (2016) (see “Materials and Methods” for the details), but with all of the 54 trees, which contain the clade (*Cocos nucifera* plus *Elaeis guineensis*) have been removed from the input forest before analysis.

The 15,9074 bp alignment of complete plastomes of palms (Arecales), Dasypogonales, and *Typha latifolia* (Poales, Typhaceae) (Fig. 8) has been downloaded from the online supplement of Barrett et al. (2016: their Fig. 4 and Fig. S5).

**Figure 9:**
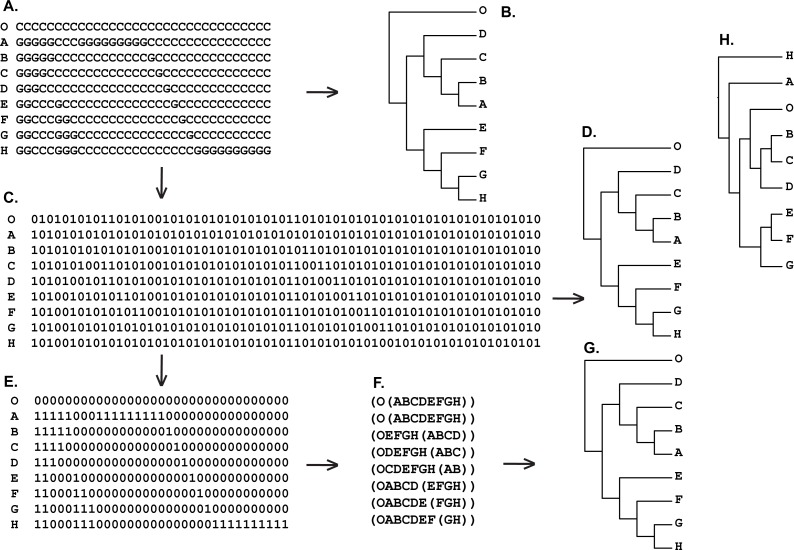
(A) Hypothetical DNA matrix from Kluge & Farris (1999: p. 206); (B) tree resulted standard MP analysis of hypothetical DNA matrix from Kluge & Farris (1999: p. 206) (length = 33, CI = RI = 1.0000); (C) binary recoding of hypothetical DNA matrix from Kluge & Farris (1999: p. 206)) (A); (D) tree resulted standard MP analysis of the binary matrix (C) (length = 66, CI = RI = 1.0000); (E) reduced binary matrix (C) with only characters that corresponded to the value of zero of the outgroup taxon (O) have been saved; (F) Hennigian Forest of the reduced binary matrix (E). “Additional…” FORESTER’s output tree file (see “Materials and Methods” for the details) selected for future analysis; (G) tree resulted standard MP analysis of the reduced binary matrix (E) (length = 33, CI = RI = 1.0000); tree was a posteriori rooted relatively taxon O∖average consensus of the Hennigian Forest (F) (score is equal to zero); (H) UPGMA phenogram of the hypothetical DNA matrix (A) (see also Kluge & Farris, 1999: p. 206).

The binary representations of the genomic (DNA) data (Figs. 6, 8 and 9) have been made by script *1001* (Mavrodiev, 2015a). In theory, different binary representations of the DNA alignments (Mavrodiev, 2015a) can be chosen as sources for the Hennigian trees. But in this paper, for future analyses, we decided to select the simplest binary matrices that resulted the “presence–absence” recoding (reviewed in Kitching et al. 1998) of the DNA alignments (Mavrodiev, 2015a), but saving the only characters that corresponded to the value of zero of the outgroup taxon. Script *1001* (Mavrodiev, 2015a) is the easiest way to generate such binary matrix. However, it can be prepared even manually using the text editor.

The Average Consensus tables are calculated following the default settings of Clann version 4.1.5 (http://chriscreevey.github.io/clann/) (Creevey, 2004; Creevey & McInerney, 2005; Creevey & McInerney, 2009) for the **avcon** command with the branch lengths of the all input trees assigned to unity (Creevey, 2004) and also used as future inputs for PAUP* 4.0b10 (Swofford, 2002) (PAUP* hereinafter). All input-trees are treated as weighted equally.

In most of the analyses the optimality criterion of the best tree is defined in the simplest way—as a distance with non-weighted least squares (Creevey, 2004; Creevey & McInerney, 2005; Creevey & McInerney, 2009; Swofford, 2002). In the case of the analysis of the Hennigian forest of Matrix Z (Farris, 1997) (Fig. 5E–5H) we used the weighted least squares (exponential weights with *P*′ = 7 (Swofford, 2002)). Different formal criteria, such as “Balanced Minimal evolution” (BME) (Desper & Gascuel, 2002; Swofford, 2002) (Figs. 7B and 7C) can also be used.

All standard Maximum Parsimony (MP) analyses as well as Maximum Parsimony Bootstrap (MP BS) estimation (Fig. 6) have been conducted in PAUP* (Swofford, 2002), sometimes (Figs. 7 and 8) as implemented in CIPRESS (Miller, Pfeiffer & Schwartz, 2010) using 1,000 random addition replicates (saving no more than 100 trees per replicate), and with the TBR branch swapping/MulTrees option in effect; the gaps were treated as “missing entities”.

The routine manipulations with the matrices and the tree-files were performed with Mesquite v. 3.01 (Maddison & Maddison, 2011), PAUP* (Swofford, 2002) and FigTree v. 1.4.2 (Rambaut, 2012).

## Results and Discussion

In Cladistics, clades must be based solely on “derived” or apomorphic character-states (e.g., Hennig, 1966; Platnick, 1985; Nelson, 2004; Williams & Ebach, 2008). For example, in the case of the binary matrix, where the character state “zero” is defined as “plesiomorphic” before the analysis, all clades should be based solely on the state “one” (e.g., Platnick, 1985). However, today the 3TA (Nelson & Platnick, 1991) is the only method, which completely avoids grouping on plesiomorphy (e.g., Williams & Ebach, 2008). Therefore, analyzing all of the selected matrices (Figs. 1–9) we have followed the general logic of 3TA. Specifically, (1) we tried to explicate all possible Hennigian trees *a priori* to the analyses in order (2) to find the best-fitting trees as the next step.

Five out of the seven binary matrices-examples (Figs. 1, 3, 4, 5) we analyzed have been established as a main sources of the major empirical claim against 3TA (and in our mind, against the whole idea of grouping solely on synapomorphies)—the principle inability of this method to operate with the putative reversals (Kluge, 1994; Farris, 1997; Farris & Kluge, 1998 reviewed in Siebert & Williams, 1998, Mavrodiev, 2015b and Mavrodiev, 2016). Using these critical examples we are demonstrated (Figs. 1, 3, 4 and 5) that this claim is not relevant to our recent proposal. Also using the original critical example of Kluge & Farris (1999) we also showed that our method could successfully handle the non-clock molecular data (Fig. 9).

We are not viewing our recent approach as an alternative to 3TA. However, there are two major differences between our recent proposal and the 3TA as originally designed and implemented by Nelson & Platnick (1991). (1) The 3TA deals with the three-taxon statements (3TS) or *minimal* relationships (Williams & Siebert, 2000; Williams & Ebach, 2006; Williams & Ebach, 2008). The set of 3TSs must be established as a binary matrix and used as an input for the parsimony program (Nelson & Platnick, 1991; see also Williams & Siebert, 2000, Williams & Ebach, 2006. Williams & Ebach, 2008 and Mavrodiev & Madorsky, 2012).

In this paper, we operate directly with *maximal* relationships (Nelson & Platnick, 1981; summarized in Williams & Ebach, 2006 and Williams & Ebach, 2008) *written just as trees*, not as binary matrices, while also using the Average Consensus method instead of the MP analysis.

Dealing with relations, not with actual “data”, clearly distinguishes our approach from all optimization-based methods, both the standard alignment-based methods and the “direct” alignment-free approaches (e. g., Wheeler, 1996; Wheeler, 2001).

In summary:

(1) Script FORESTER helps to rewrite every character of the binary matrix as a tree in a Cladistics way basing all of the groups solely on *a priori* defined apomorphic character-state “1”.

(2) Next, the forest of these “maximal” Hennigian trees can be used to calculate their average consensus (Lapointe & Cucumel, 1997; Lapointe & Levasseur, 2004) (Figs. 1–9).

A possible issue of the Hennigian approach to the data is the inability to operate with the putative reversals (reviewed in Farris & Kluge, 1998, Siebert & Williams, 1998 and Mavrodiev (2015b), unless, however, the ‘reversals’ are scored as separate apomorphic character-states before the analysis (Mavrodiev, 2015b).

Paradoxically, the Average Consensus method, if applied to the Hennigian forests, helps to identify the ‘reversal’-based clades without the separate scoring of the putative reversals, or, in other words, *despite* the absence of evidence from the primary data (Figs. 1, 3 and 4). A similar effect had been described by Nelson & Platnick (1991) for 3TA and discussed in more detail by Siebert & Williams (1998) as well as by Mavrodiev (2016) who named this paradox as a “Synthetic Theorem of Nelson and Platnick”.

For example, consider the modified Matrix 17 from Nelson & Platnick (1991) (Fig. 1). According to the MP optimization, group (EF) is based on the plesiomorphic character-state zero and therefore may be treated by someone as a putative ‘reversal’-based clade (e.g., Farris, 1997). However as explained above, grouping based on the plesiomorphic character state is prohibited within the Hennigian framework (e.g., Platnick, 1985; Nelson, 2004; Williams & Ebach, 2008). Therefore the standard MP solution for the group (EF) is clearly non-Hennigian or phenetic (e.g., Williams & Ebach, 2008; Mavrodiev, 2015b).

*None of the trees from the array c. (*Fig. 1*) contains any plesiomorphic-based groups*, *but clade (EF) is still defined in the average consensus tree* (Fig. 1) *throughout the analysis of forest c.* (Fig. 1) or, in other words, *despite the lack of evidence from the primary data* (see also Nelson & Platnick, 1991: 363 for the similar discussion). The same situation is detected in the cases of Table 1 from Kluge (1994) (Fig. 3), and Table 5 from Kluge & Farris (1999) (Fig. 4).

The Average Consensus technique, if applying to the Hennigian forests, may also help to avoid more potentially negative effects of putative reversals. For example, regarding his Table 3, Kluge (1994: 408–410) mentioned that taxa G and F are highly supported sisters with taxon G exhibiting reversals only in characters one and two. As discovered by Kluge (1994), the 3TA (Nelson & Platnick, 1991) removed taxon G from F, despite the strong evidence of their relationship. A similar situation appears in the case of Matrix Z, designed by Farris (1997) simply by duplication of the Table 3 from Kluge (1994) (Farris, 1997; Siebert & Williams, 1998).

However, the tree-shapes of the average consensuses of the Hennigian forests of the Table 3 from Kluge (1994) (Fig. 5A–5D) and Matrix Z from Farris (1997: 136) (Fig. 5E–5H) appeared to be identical to the topologies, which were the result of the MP analyses of Table 3 and Matrix Z (Kluge, 1994; Farris, 1997). 3TA, with fractional weighting procedure (Nelson & Ladiges, 1992; Williams & Ebach, 2008) can also compensate the negative effect of putative reversals in the same situations (Siebert & Williams, 1998).

Like the 3TA (Nelson & Platnick, 1991), the Average Consensus analysis of the forests of maximal relationships can successfully recognize groups for which the standard optimization criteria of the MP analysis produce no unequivocal synapomorphies (Nelson, 1996; Williams & Ebach, 2005) (Fig. 2). For example, group (BCD) is successfully recognizable after the Average Consensus analysis of the three trees, each representing the conflicting binary characters from Matrix 1 from Nelson (1996) (see also Williams& Ebach, 2005; Williams & Ebach, 2006) (Fig. 2).

Williams & Ebach (2006: 414) offered a very similar solution for the clade (BCD). These authors mentioned that *it would seem more appropriate to re-write the characters of Matrix 1 from Nelson (1996) in a tree-form representing the relationships exactly*, such that the three “characters” are AD(BC), AC(BD) and AB(CD), which, when “*combined”* (Williams & Ebach, 2006: 414, italics are ours), unambiguously provide evidence for the solution A(BCD) (Williams & Ebach, 2006: 414). In contrast to this intuitively clear solution, as well as to the results of the Average Consensus analysis (Fig. 2), neither of strict, majority rule, Adams’, or combinable component consensuses (see Kitching et al., 1998 and Swofford, 2002 for the reviews and implementation of the methods) of the three trees AD(BC), AC(BD), and AB(CD) (Williams & Ebach, 2006: 414) are able to recognize the group (BCD).

Williams & Ebach (2006: 414) are also noted that the re-writing of the binary characters effectively converts an ordinary (phenetic) matrix into a “Cladistic matrix”. In our mind, a Cladistic matrix is similar, but still not of the same entity as a simplest forest of the Hennigian trees (see Siebert & Williams, 1998: 242 for the initial elementary examples of the Cladistic matrices called by these authors as a “tabular formulations of the 3TA” of the conventional data; see also Williams & Ebach, 2006: 412, Nelson & Ladiges, 1992: 492–493 and Williams & Ebach, 2008).

In the first approach, the simplest options of the search (the least-square criteria of the fit plus un-weighted algorithms) should be sufficient to estimate the reasonable average consensuses. However, the Average Consensus approach allows various schemes of weighing as well as different optimization criteria (Creevey, 2004; Lapointe & Levasseur, 2004). For example, the use of the exponential weights of the least squares as implemented in PAUP* (Swofford, 2002) or BME-algorithm (Desper & Gascuel, 2002; see (Swofford, 2002 for recent implementation) may increase the efficiency of the search (Figs. 5 and 7).

The proposed approach may also handle the clock-like (e.g., Fig. 6) or non clock-like matrices (e.g., Fig. 9), as well as the hypothetical or the real data, either the DNA sequence data or the morphological characters (Figs. 6–8). There are several possible ways to do this. As a tentative test, for someone it may seem to be necessary to re-recode the conventional multistate matrices as a binary matrices, to establish these matrices as a Hennigian forests, and to estimate the average consensus trees that fit these Hennigian forests (Figs. 6–8). Therefore, even in the cases of the big molecular data (Fig. 8), the taxon-character matrices (molecular alignments) may be necessary only as sources of the arrays of maximal relationships.

We also would like to stress, that for the real data it is also true that at least in some cases the clades may appear within the final tree without the direct evidence from the primary data. For example, the clades (*Homo* plus *Pan*) or (*Cocos nucifera* plus *Elaeis guineensis*) are still recognizable by the Average Consensus method, even if all of the trees from the corresponding Hennigian arrays, that contain the same clades (*Cocos nucifera* plus *Elaeis guineensis*) or (*Homo* plus *Pan*), will be excluded from the forest-inputs (Figs. 6C, 7C and 8C). In other words, the clades (*Homo* plus *Pan*) or (*Cocos nucifera* plus *Elaeis guineensis*) appears within the final average consensus trees (Figs. 6–8) as a kind of *relations* (e.g., Nelson, 2011), rather than the character-based entities.

## Conclusions

1. Binary matrix with *a priori* defined plesiomorphic character-state may be re-written as a simple set of rooted branching diagrams (as a “Hennigian forest”).
2. The last might be analyzed by the Average Consensus method.
3. This procedure eventually avoids the taxon-character matrix from the analysis of the data. Within the proposed framework, the ordinary (phenetic) matrix is necessary only as a “source” of the Hennigian trees, not as a primary subject of the analysis.
4. Within this approach, the criteria of the best trees based on the character-state changes are not required.
5. Dealing with the relations, not with the actual “data”, clearly distinguishes our approach from all optimization-based techniques, either the standard taxon-character alignment-based methods, or the “direct” alignment-free method.
6. The Average Consensus method, if applied to the Hennigian forests, may helps to identify the clades *despite* the absence of the direct evidence from the primary data. Because of such ability, the ‘reversal’-based clades can always be found by this approach without the separate scoring of the putative reversals.

##  Supplemental Information

10.7717/peerj.3578/supp-1 Supplemental Information 1 Tables, matrices and trees for Figs. 1–9.

## Additional Information and Declarations

### Data Availability

The following information was supplied regarding data availability: GitHub: https://github.com/dellch/forester.
